# Letter to the editor: Just a coincidence? Two severe human cases due to swine influenza (SIV) A(H1N1)v in Europe, October 2016

**DOI:** 10.2807/1560-7917.ES.2017.22.10.30478

**Published:** 2017-03-09

**Authors:** Cornelia Adlhoch, Pasi Penttinen

**Affiliations:** 1 European Centre for Disease Prevention and Control, Solna, Sweden

**Keywords:** Influenza, zoonotic infections, swine A(H1N1)v

**To the editor: **We read with concern the reports of two human cases infected with swine influenza virus (SIV) that both occurred in October 2016 in Europe [1,2]. One case was a school-aged child in the Netherlands with history of eczema, while the other was a middle-aged man in Italy with obesity as underlying condition. The similarities of these cases were striking: both developed severe respiratory symptoms with a rapid progression, finally requiring admission to intensive care and use of extracorporeal membrane oxygenation (ECMO). Antiviral treatment was initiated in both cases more than three days after onset of symptoms, and only after the symptoms worsened, invasive mechanical ventilation and ECMO support were initiated. Both cases recovered and were discharged from hospital. 

Eurasian avian-like SIV A(H1N1)v were identified in both patients. In Europe, these viruses circulate widely in the pig population [3]. The viruses isolated from the patients differed genetically from each other. Both cases had presumably visited pig farms before onset of symptoms, suggesting direct contact or indirect exposure to infected pigs, e.g. through contaminated surfaces or via aerosol. In both cases, an initial positive influenza A detection with subsequent inconclusive typing results was followed by whole genome sequencing, which identified the swine virus origin. The same virus was also detected in the pigs at the visited farm in the Netherlands. 

These two cases are the first severe human cases of SIV A(H1N1)v reported to the European Centre for Disease Prevention and Control (ECDC) or the World Health Organization Regional Office for Europe (WHO/Europe) since the 2009 influenza A(H1N1) pandemic, apart from a few sporadic detections in mild or asymptomatic persons during research projects [4]. The detection of these cases may have benefited from the availability of whole genome sequencing for specimens with inconclusive results or nontypeable influenza. That reports of human cases in Europe are rare is in contrast to the more frequent sporadic reports of human cases caused by SIV A(H1N1)v, A(H1N2)v or A(H3N2)v in the United States (US) and highlights the continuous possibility of spill-over of influenza viruses from swine to humans [5]. This is probably a result of the fact that more individuals in the US are exposed to infected animals because of the popularity of regular local and state fairs and agricultural exhibitions with ca 150 million visitors each year where swine are openly accessible for the visitors [6]. Because there is no continuous surveillance of influenza viruses in pigs in Europe, data mainly derive from research projects that may only provide a fragment of the overall picture. Especially little is known about the impact of trade-related network and transport structures between pig producers that may contribute to a rapid spread of new influenza viruses across Europe [3].

The 2009 pandemic was the latest of several pandemics caused by a swine-origin influenza virus [7]. The two recent human cases of swine influenza should serve as a reminder that zoonotic transmission events from pigs to humans, causing severe illnesses, can happen in Europe as well. They should also raise awareness of the need for cautious SIV case management, particularly during the seasonal influenza epidemic, in order to avoid re-assortment events between swine and human viruses and to detect any human-to-human transmission as early as possible.

People who develop influenza-like symptoms after exposure to pigs should be promptly assessed for influenza infection. Specimens should be characterised virologically and isolates should be shared with the WHO Collaborating Centres. Early treatment with neuraminidase inhibitors should be considered and patients isolated to reduce risk of further transmission, in line with relevant national recommendations. Follow-up of other exposed individuals and contacts of the index cases should be considered to identify further cases and to detect any human-to-human transmission of the virus. A rapid sharing of such information nationally as well as internationally through the European Union’s Early Warning and Response System (EWRS), the International Health Regulations [7] or directly contacting the respective authorities is a prerequisite for early identification of new emerging pandemic threats and initiation of containment and prevention measures.
